# Solitary fibrofolliculoma of the upper eyelid in a 68-year old female: a case report

**DOI:** 10.1186/s12886-020-01366-4

**Published:** 2020-03-11

**Authors:** Wenqiu Wang, Jinwei Cheng

**Affiliations:** Department of Ophthalmology, Shanghai General Hospital, Shanghai Jiaotong University School of Medicine, Shanghai, 200080 China

**Keywords:** Solitary fibrofolliculoma, Eyelid tumor, Diagnosis, Surgery

## Abstract

**Background:**

Fibrofolliculoma is a benign, perifollicular, connective tissue tumor, and it usually arises in the form of multiple lesions, but rarely as a solitary lesion. We report a case of solitary fibrofolliculoma on the eyelid.

**Case presentation:**

A 68-year-old female presented with an asymptomatic mass on the right upper eyelid. The lesion appeared as a flesh-colored, dome-shaped, smooth nodule being the size of 5 × 5 × 4 mm, with eyelashes protruding from the surface, and located on the upper lid margin. Shave excision was performed, and the diagnosis of fibrofolliculoma was confirmed finally through histological exam.

**Conclusions:**

Solitary fibrofolliculomas rarely arises on the eyelid. However, it should be suspected when a flesh-colored and doom-shaped lesion of the eyelid is encountered. The benign tumor on the lid margin can be removed by shave biopsy.

## Background

Fibrofolliculoma usually is a clinically asymptomatic multiple connective tissue tumor, appearing perifollicular, skin-colored and located on the head or neck. Multiple fibrofolliculomas generally are inherited as an autosomal dominant trait and share clinical characteristics of Birt-Hogg-Dubé (BHD) syndrome, which is associated with multiple fibrofolliculomas, acrochordons, trichodiscomas, and internal neoplasms [1, 2].

Fibrofolliculoma, rarely presents as a solitary lesion, since being firstly reported in 1984 [3]. Solitary forms are usually unassociated with other cutaneous abnormalities with typically nonhereditary [4]. Only 12 cases have, to date, been previously published in the literature [3–10]. To our knowledge, our case is the second one reported occurrence on an eyelid [5]. Herein, we presented the clinical features and surgical treatment of a rare solitary fibrofolliculoma located on the upper eyelid of a 68-year-old female.

## Case presentation

A 68-years-old Mongoloid woman presented with an asymptomatic, flesh-colored lesion on the right upper eyelid. The lesion had slowly increased in size over 5 years. No similar lesions were found on other parts of the body. Her medical and family histories were unremarkable, and she had experienced no triggering trauma.

**Fig. 1:**
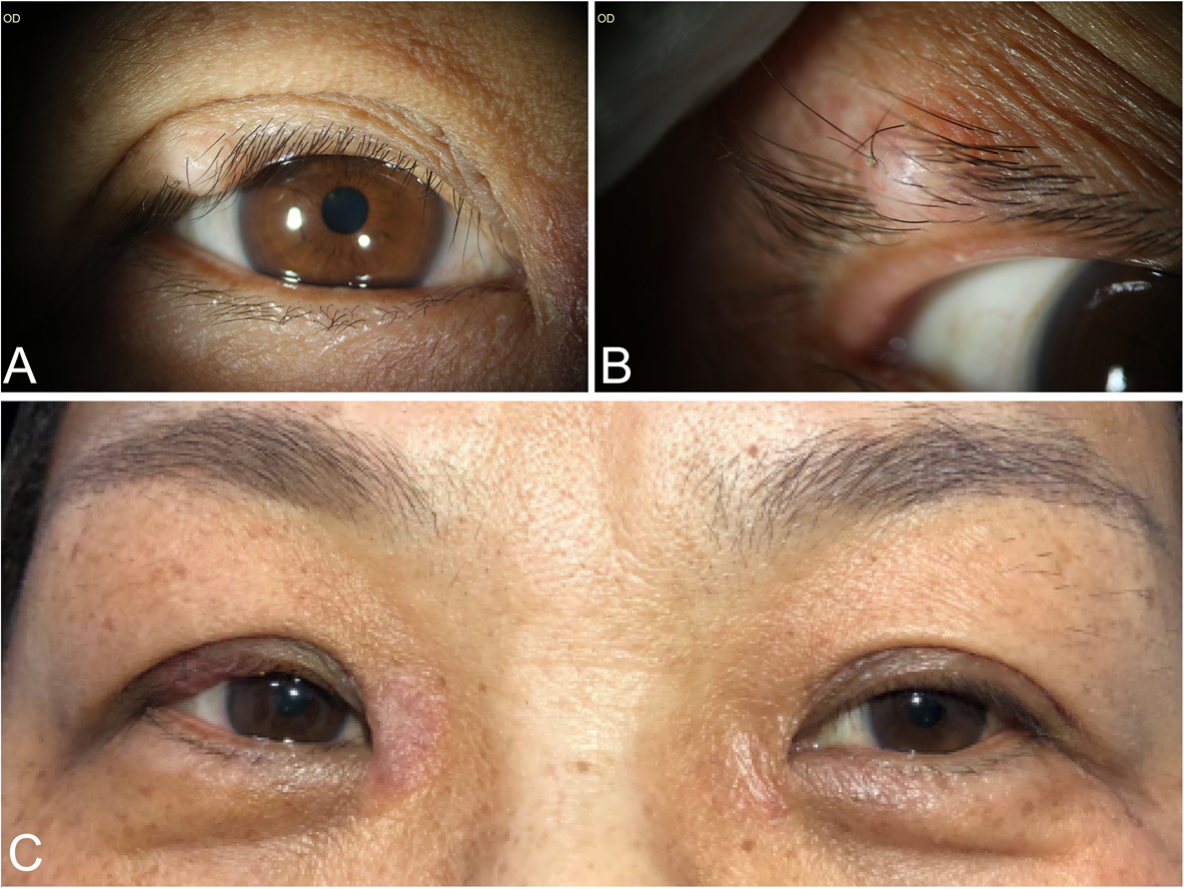
Preoperative and postoperative appearance of the right eye. **a** A solitary skin-colored, dome-shaped lesion locates on the right upper eyelid. **b** The lesion locates on the anterior lamella of the lid margin, with several eyelashes protruding from its smooth surface. **c** Two weeks postoperatively, the upper lid maintained its shape

Upon ophthalmologic examination, the protruding lesion was found to be approximately 5 × 5 × 4 mm and located on the upper lid margin (Fig. 1a). Palpation of the lesion did not elicit pain, and the lesion was non-slidable. On the photography of anterior segment, the nodule was verified as flesh-colored, dome-shaped, with eyelashes on the smooth surface, and the lesion located on the anterior lamella of the lid margin, without superficial ulceration and dilated blood vessels. (Fig. 1b). The conjunctiva, cornea, and lens were unremarkable and so as the fundus examination results. Examinations showed that the left eye was normal. The visual acuity of both eyes was 20/20.

The lesion was removed by shave excision under local anesthesia. The lesion was non-slidable, and it was adherent to the tarsal plate and its covered skin. Anterior lamella of the eyelid was resected with a trigonal wedge, with the removal of 1 mm of extra tissue from the margin of the lesion, and the thin layer of the tarsal plate. The anterior lamella defect of the upper eyelid was repaired using A-T flap. A gray line split was performed on the cut ends of skin defects, then, the skin defects were sutured directly. We checked the preauricular lymph nodes, and no lymphadenopathy was found. After the operation, the patient was compression bandaged for 24 h.

**Fig. 2:**
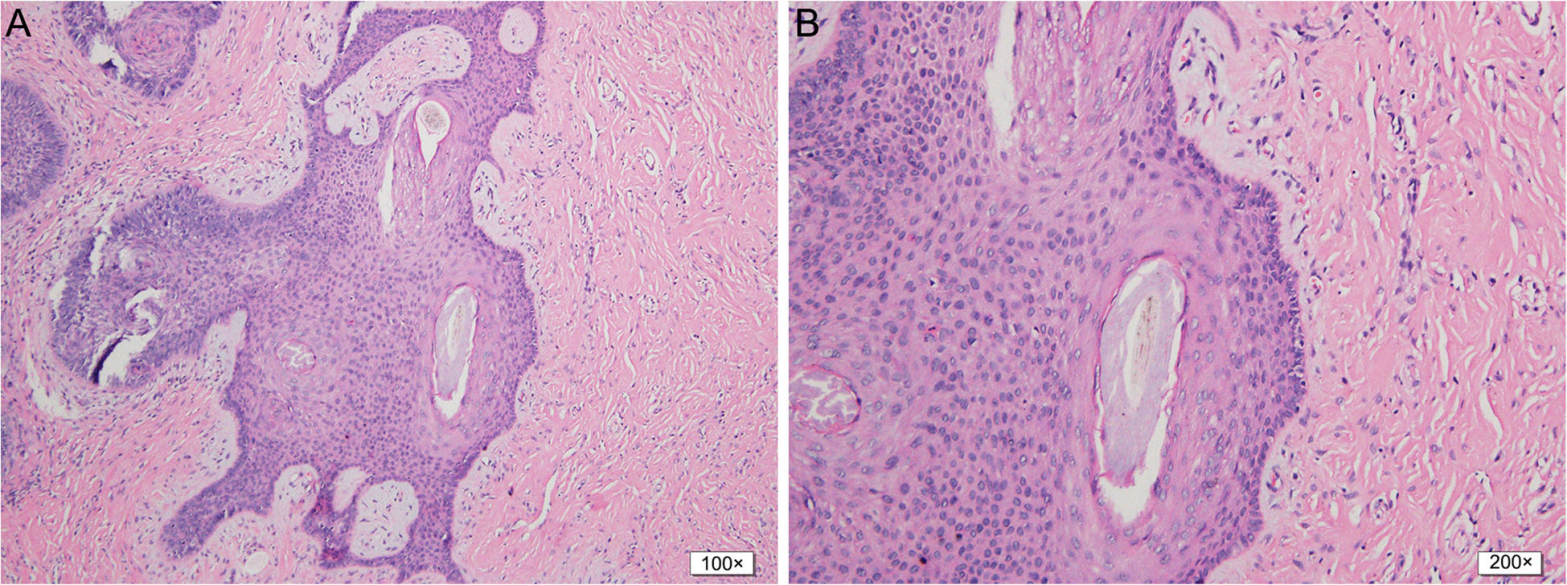
Histopathological photograph of the solitary fibrofolliculomas. **a** Low-power photograph (100×) shows the proliferation of multiple thin strands of basaloid cells extending from the central follicle into the surrounding fibrous stroma. **b** High-power photograph (200×) shows the fibrous stroma contents with mucin, surrounding the hair follicle

Histologically examination of the lesion showed a well-defined tumor mass involving a hair follicle, and a proliferation of multiple thin strands of basaloid cells, extending from the central follicle into the surrounding fibrous stroma. The fibrous stroma presented a sharp contrast with the surrounding dermis. Hematoxylin-eosin stains contained mucin content in the stroma (Fig. 2). The histologic findings were characteristic of fibrofolliculoma.

Two weeks after the surgery, the patient had no particular complain. The right upper lid showed almost identical to that of the left (Fig. 1c). During the 3-month follow-up, no signs of recurrence or new lesions appeared.

## Discussion and conclusions

The cases of solitary fibrofolliculoma are extremely rare, since being first reported in 1984 [3]. Only eight prior papers, involving 12 cases, have previously been published worldwide [3–10]. We reviewed the previously published cases and found that most lesions were found on the face, with only 2 around the eyes, one on the eyelid and the other on the eyebrow (Table 1). Among all 13 cases, including the present one, solitary fibrofolliculoma occurs more frequently in women (5 men and 8 women), with the mean age at presentation being 51 years. The duration of symptom varying from several months to many years. Solitary fibrofolliculomas share the clinical appearance of multiple fibrofolliculomas, yellowish to flesh-colored, dome-shaped papules.

**Table 1:** Summary of previous reported cases of solitary fibrofolliculoma

Author	Country	Year	Age (years)	Sex	Duration of symptom	Location	Size of tumor	Mass appearance	IHC features	Previous clinical diagnosis	Treatment	Recurrent
Sohn KM [10]	Korea	2018	50	M	3 years	right posterior auricular area	10 ×12 mm	slightly pruritic, flesh-colored			shave biopsy	
Criscito MC [9]	US	2017	72	F	several years	Left cheek	4-mm in diameter	dome-shaped flesh-colored papule			excision	
Cho E [8]	Korea	2012	45	M	3 years	ear	12×10×8mm	flesh-colored mass			shave biopsy	No (4 month follow-up)
Cesinaro AM [7]	Italy	2010	63	F	several months	nose	12×10 mm	skin-colored, smooth surface	co-expression of CD34 and factor XIIIa	basal cell carcinoma	excision	No (6 month follow-up)
Chang JK [5]	Korea	2007	37	F	1 year	eyelid	5×5 mm	skin-colored bean-sized mass		chalazion	excision	No (2 year follow-up)
Hong JK [6]	Korea	1997	40	F	1 year	left parietal scalp area	7×6×5mm	Skin-to-pink colored protruding mass with a shallow central dell			excision	
Starink TM [4]	US	1987	49	M	2 years	chin	5mm in diameter	Yellowish nodule		epiderrnoid cyst	biopsy	
			20	F		nose		skin-colored papule		fibroma		
			50	M	1 year	left cheek		skin-colored papule		intradermal nevus		
			60	F	Several years	ear	3 mm in diameter	domeshaped papule with a central comedolike opening		epiderrnoid cyst		
			52	M		eyebrow	6×4 mm			epidermoid cyst		
Scully K [3]	Canada	1984	62	F	4 month	chin	5mm in diameter	skin-to-pink-colored, dome-shaped papule			excision	

Histologically, fibrofolliculoma has both an epithelial and a mesenchymal origin, showing distinctive and characteristic features with minor variation [3]. The center of the lesion presents a hair follicle and consists of an expansion of the fibrous root sheath, which typically surrounds the hair follicle, along with proliferating bands or ribbons of perifollicular connective tissue. Cesinaro and coauthors found immunohistochemical expression of factor XIIIa in the bizarre perifollicular cells in a background of CD34-positive spindle cells, which aids for better characterize the nature of the lesion [9].

Since solitary fibrofolliculoma is extremely infrequent and definitively diagnosed only by histological results, it can be easily overlooked or clinically misdiagnosed. The 37-year female patient reported by Chang and coauthors was previously misdiagnosed as chalazion and received incision and curettage only. For years, her condition had not improved, recurring several times [5]. Fortunately, fibrofolliculoma rarely develops to malignancy. Once this type of lesion in the eyelid is observed, a diagnosis of fibrofolliculoma should be considered. As the lesion of the presented case was located on the eyelid, it also should be included as a differential diagnosis from malignant conditions such as basal cell carcinoma and squamous cell carcinomas, which are the most common malignant eyelid tumors. Basal cell carcinomas appear as a translucent, waxy papule with a rolled, pearly border, and telangiectasia. As it enlarges, central ulceration usually develops. Squamous cell carcinomas appear as a painless, elevated, nodular, or plaque-like lesion with chronic scaling and fissuring of the skin. The characteristic features of squamous cell carcinomas also include pearly irregular borders and a tendency to develop ulceration with irregular rolled edges.

Sharing the characteristics of BHD syndrome, fibrofolliculoma is considered to be hamartomas, composed of both connective tissue and follicular epithelial components [11]. Most fibrofolliculoma may have some common histogenesis such as abnormal function of hair follicle bulge cells, and differential diagnosis should be considered in the histopathological exam.

Surgical excision is usually chosen for the skin fibrofolliculomas in the first operation for pathological diagnosis. The CO_2_ laser, or erbium-doped YAG laser, might be a better choice for multiple fibrofolliculomas or recurrent lesions [12, 13]. Currently, there are no uniform standards for eyelid lesions. Surgical treatment for an eyelid lesion should be individualized based on the size, growth rate, invasion, and interference with eyelid function and aesthetics [14, 15]. Benign eyelid lesions can be treated with less invasive techniques, such as shaving biopsy, and simple excision. For the benign tumors on lid margin, shave excision including a 1-mm margin of normal tissue should be considered [16, 17]. Also, postoperative follow-up is necessary to demonstrate early recurrence, and to improve facial appearance as required.

We have presented a rare case of the flesh-colored and doom-shaped lesion arising on the eyelid and show the histologic features of fibrofolliculoma. Although rare, a diagnosis of solitary fibrofolliculoma may be considered when a similar lesion is observed. As the benign tumor on the lid margin, shave excision including a 1-mm margin of normal tissue should be considered. Our report highlights both the clinical and the histopathological features, which are important for appropriate treatment and prognosis prediction.

## Data Availability

More data, if necessary, is available from the corresponding authors upon reasonable request.

## Abbreviation

BHD syndrome: Birt-Hogg-Dubé syndrome
